# TSPO imaging in parkinsonian disorders

**DOI:** 10.1007/s40336-016-0171-1

**Published:** 2016-04-11

**Authors:** Alexander Gerhard

**Affiliations:** Wolfson Molecular Imaging Centre, Institute of Brain, Behaviour and Mental Health, The University of Manchester, 27 Palatine Road, Withington, Manchester, M20 3LJ UK

**Keywords:** Neuroinflammation, Microglia, TSPO, Parkinson’s disease, [^11^C]-(*R*)-PK11195- PET

## Abstract

Microglial activation is a key aspect of the neuroinflammatory process in neurodegenerative disorders including idiopathic and atypical parkinsonian disorders. With positron emission tomography (PET) it has become possible to image this phenomenon in vivo and over the last years patterns of microglia activation corresponding with the known distribution of neuropathological changes in these disorders have been demonstrated using this technique. In addition the effects of interventions aimed at suppressing microglia activation as part of interventional trials have successfully been demonstrated. Current research aims at evaluating PET tracers for microglial activation with more favorable properties than the prototypical [^11^C]-(*R*)-PK11195, as well as developing tracers targeting additional parameters of the neuroinflammatory process like astroglial function.

## Introduction

Inflammation is a reaction of living tissues to injury and acute and chronic inflammation can be distinguished. Acute inflammation comprises the immediate and early response to an injurious agent and is basically a defensive response that paves the way for repair of the damaged site. Chronic inflammation results from stimuli that are persistent. In the periphery, inflammation consists of leukocytic infiltrates characterized by polymorphonuclear cells (neutrophils) and mononuclear cells (macrophages, lymphocytes, plasma cells).

Over the last years, the term “neuroinflammation” has come into widespread use. This term generally denotes chronic, CNS-specific, inflammation-like glial responses that do not reproduce the classic characteristics of inflammation in the periphery but that may cause and/or sustain neurodegenerative events including plaque formation, dystrophic neurite growth, and excessive tau phosphorylation [1]. Neuroinflammation has been implicated in chronic unremitting neurodegenerative diseases such as Parkinson’s and Alzheimer’s disease; diseases that historically have not been regarded as primary inflammatory disorders. This new understanding has come from rapid advances in the field of microglial and astroglial neurobiology over the past 15–20 years. These advances have led to the recognition that glia, particularly microglia, respond to tissue insults with a complex array of inflammatory cytokines and actions, and that these actions transcend the historical vision of phagocytosis and structural support that has long been described in the term “reactive gliosis.” Microglia are now recognized as the prime components of an intrinsic brain immune system and as such they have become a main focus in cellular neuroimmunology and neuroinflammation [1].

## Microglia

Microglia are normally quiescent, brain macrophages and represent the resident immuno-competent cells of the central nervous system. Recent fate mapping studies have shown that under your homeostatic conditions microglia are not derived from the bone marrow but from haematopoietic stem cells in the yolk sac during embryogenesis [2]. Del Rio Hortega [3] first recognized the pathological importance of microglia in the central nervous system (CNS), and he also coined the name. They constitute up to 10 % of the total cell population of the CNS. In conditions of intact blood–brain barrier when blood-borne cells are largely absent, microglia together with perivascular cells are the first line of the brain’s immune defense system. Any neuronal insult or damage induces an activation of resting microglial cells. Activated microglia produce a variety of proinflammatory molecules, change their morphology and, if cell death occurs, finally mature into full-blown macrophages [4, 5].

This morphological change has been well documented in the facial nerve transection model, where microglial activation can be assessed in an environment without blood–brain barrier disruption or migration of new bone marrow-derived macrophages. The model demonstrates the capacity of resident microglia to undergo morphological changes and activation with expression of new surface markers and to proliferate around motor neurons of the facial nucleus [6].

Microglia activation in vivo occurs as a graded response. The transformation of microglia into potentially cytotoxic cells is under strict control, mainly in response to neuronal or terminal degeneration, or both. Activated microglia are mainly scavenger cells but also perform various other functions in tissue repair and neural regeneration [7]. They form a network of immune alert resident macrophages with a capacity for immune surveillance and control. Activated microglia can destroy invading micro-organisms, remove potentially deleterious debris, promote tissue repair by secreting growth factors and thus facilitate the return to tissue homeostasis. An understanding of intercellular signaling pathways for microglia proliferation and activation could form a rational basis for targeted intervention on glial reactions to injuries in the CNS; for an extensive review on microglia see [7–10].

## Activated microglia and neuroinflammation in the neuropathology of parkinsonian disorders

### Idiopathic Parkinson’s disease

The main pathological finding in IPD (idiopathic Parkinson’s disease) is the degeneration of dopaminergic projections from the substantia nigra pars compacta to the caudate nucleus and putamen (striatum). Intraneuronal Lewy bodies and Lewy neurites composed of the protein α-synuclein are the pathological hallmarks of the disease. However, the neuropathological changes are not confined to the substantia nigra [11]. This is reflected in a staging system for IPD proposed by Braak [12]. According to Braak, the neuropathological process in PD, as represented by α-synuclein-immuno-positive Lewy neurites and Lewy bodies, first occurs in the dorsal IX/X motor nucleus and olfactory bulb (stage 1). From the medulla oblongata, it spreads into the pons and midbrain (stages 2 and 3) and eventually into the anterior cingulate, association temporal mesocortex and neocortex (stages 4–5), and finally primary cortical areas (stage 6). There is emerging evidence that the spread of the protein aggregate might follow a propagation mechanism reminiscent of human prion disease [13].

In idiopathic Parkinson’s disease activated microglia have initially been described in the substantia nigra [14] and more recently also in putamen, hippocampus, transentorhinal cortex, cingulate cortex and temporal cortex [15].

The exact contribution of microglial activation to the degenerative process in IPD is presently not well understood. There is evidence from humans [16] and monkeys [17] exposed to the nigral toxin MPTP that after a discrete insult progression of parkinsonian symptoms can occur, and this is accompanied by a prolonged neuroinflammatory response even years after the acute exposure. However, it is possible that the activation of microglia has different functions (detrimental/beneficial) at the various stages of the disease. Although neuropathologically associated with the degenerative process, it is not necessarily responsible for it.

### Atypical parkinsonian disorders

In multiple system atrophy (MSA) typical histopathological changes consist of neuronal loss in the nigrostriatal and olivopontocerebellar pathways with α-synuclein-positive, argyrophilic staining, glial cytoplasmic inclusions associated with reactive astrocytes and activated microglia [18–20] .

In PSP (progressive supranuclear palsy) there is atrophy of the brainstem, internal globus pallidus, amygdala, frontal and parietal lobe, and nigral depigmentation. Microscopically, neuronal loss is found in brainstem oculomotor nuclei, pallidum, substantia nigra, subthalamic nucleus, and frontal cortex. The pathological hallmarks of PSP are intraneuronal neurofibrillary tangles (NFTs) composed of abnormally phosphorylated microtubule-associated tau-protein and neuropil threads [21]. The cell loss in PSP is accompanied by microglial activation [22].

Gross pathological findings in CBD (corticobasal degeneration) include narrowing of cortical gyri often in a peri-Rolandic distribution. The superior frontal gyrus and inferior parietal area are usually most affected while the inferior frontal gyri, temporal, and occipital lobes are spared. The atrophy is characteristically asymmetric [23] and detailed analysis of the distribution of the microglial burden shows correlations with the anatomical regions affected in CBD [22].

As shown in these studies referenced above, the neuropathology of parkinsonian disorders is closely associated with microglial activation. The exact role of neuroinflammation in neurodegenerative disorders, however, is unclear. Post-mortem studies and animal experiments have numerous methodological limitations; for a review see [24].

In vivo imaging in man using positron emission tomography (PET) can overcome some of these, and the principles and applications are detailed below.

### PET imaging of inflammation in parkinsonian syndromes

One of the molecules expressed during microglial activation is the mitochondrial translocator protein 18 kDa (TSPO), formerly known as the ‘peripheral benzodiazepine binding site’ (PBBS) [25, 26]. This protein is predominantly located on the outer mitochondrial membrane, and has numerous functions including steroid biosynthesis and protein transport. It is distinct from the GABA A–receptor complex, the site of central benzodiazepine binding.

**Fig. 1:**
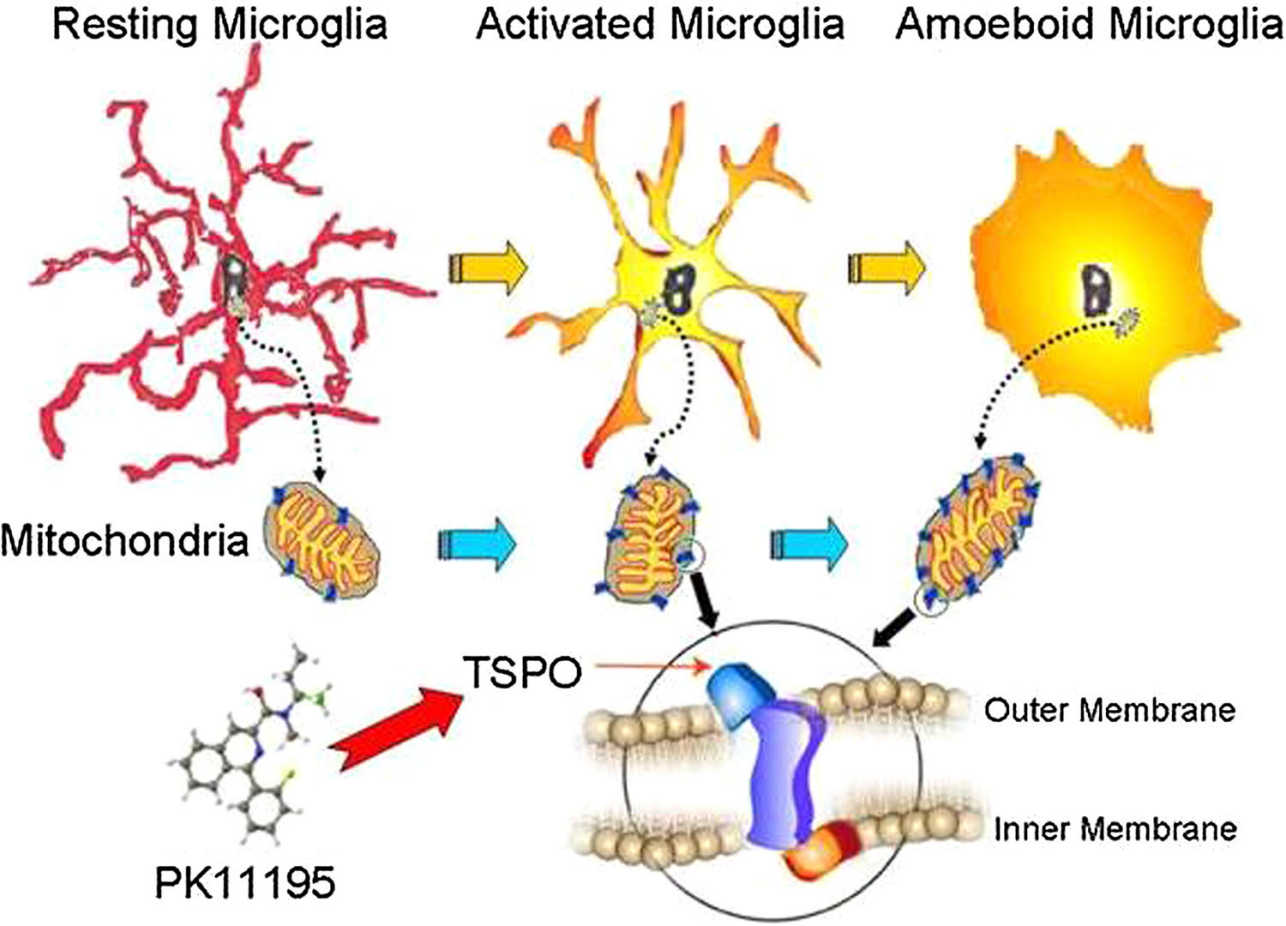
Schematic drawing of microglial activation with increased expression of TSPO in mitochondria, and binding of PK11195 to TSPO (modified after [26])

PK11195 (1-(2-chlorophenyl)-*N*-methyl-*N*-(1-methylpropyl)-3-isoquinolinecarboxamide), a highly specific TSPO ligand, exhibits low binding in the normal brain, while increased binding is indicative of increased TSPO expression in microglia following neuronal injury [27, 28] (Fig. 1). The ^11^C-labeled (*R*)-isomer of PK11195 can thus be used to image activated microglia in vivo. While [^11^C]-(*R*)-PK11195 is the best pre-clinically and clinically characterized PET ligand to image microglial activation, new, often [^18^F]-based, radiotracers for microglial activation have been developed recently, but so far have only been evaluated to a limited extent in a clinical context [29, 30].

**Fig. 2:**
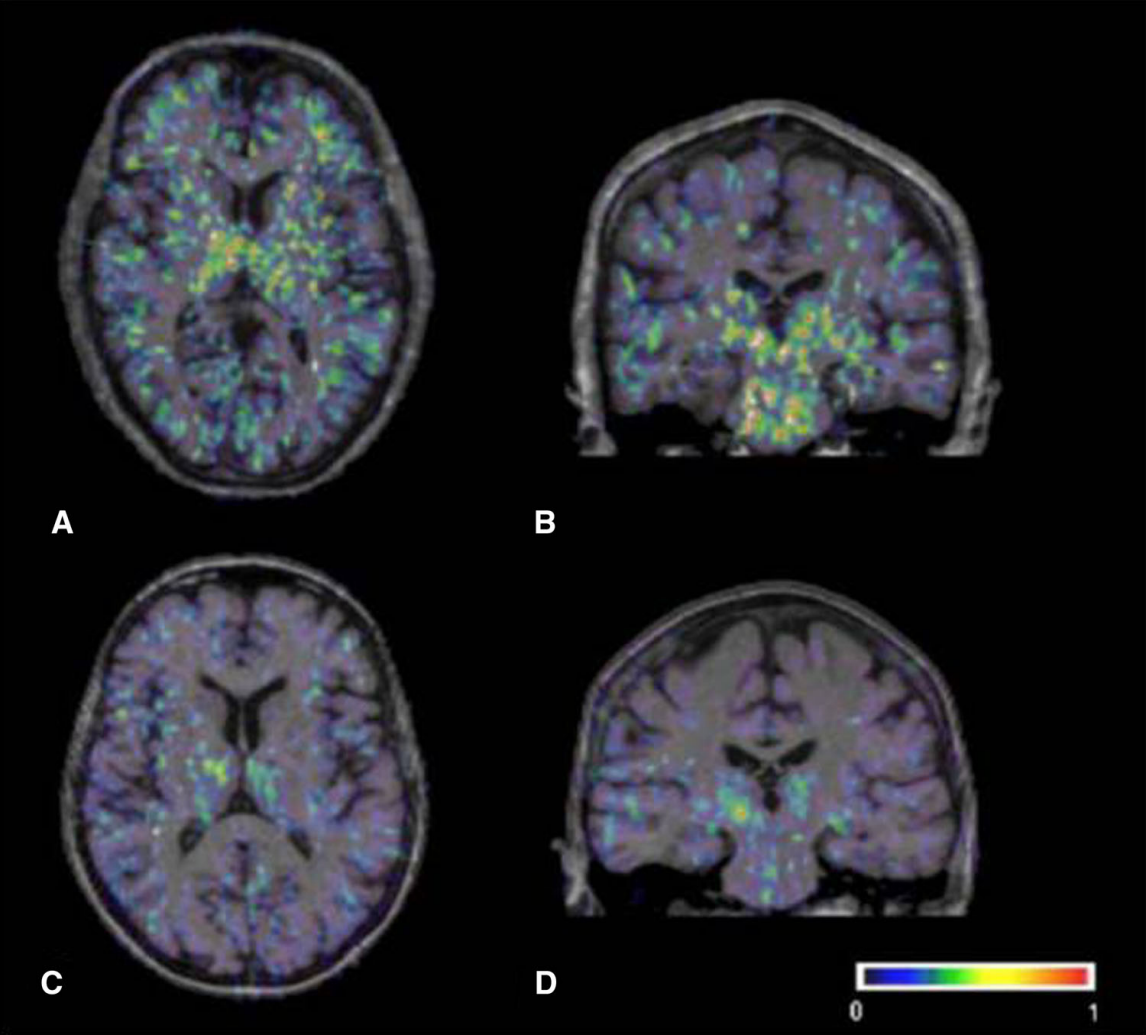
Transverse and coronal sections of binding potential maps coregistered to the individual MRI. In the PD patient (**a**, **b**), binding is increased in the basal ganglia, pons and frontal regions, while the healthy control person (**c**, **d**) only shows constitutive [^11^C](R)-PK11195. Binding in the thalamus and pons. The color bar denotes binding potential values from 0 to 1. Modified with permission from [31]

In Parkinson’s disease increased [^11^C]-(*R*)-PK11195 BP (binding potential) has been shown using ROI (region of interest) and SPM (statistical parametric mapping)-based analyses in the striatum, thalamus, pons, frontal and cingulate cortex in 18 PD patients compared to 11 controls [31] (Fig. 2). There was no correlation of the extent of microglial activation with clinical severity or findings on [^18^F]-DOPA PET. Eight of the patients were followed up longitudinally over 24 months, and, although the disease had clinically progressed, there was no significant change in the extent of microglial activation. In contrast, Ouchi and colleagues [32] studied 10 early, drug-naïve PD patients and showed increased [^11^C]-(*R*)-PK11195 BP in the midbrain, which was negatively correlated with dopamine transporter binding measured by [^11^C]-CFT PET and positively correlated with motor severity. Differences in methodology as well as motor severity (mean UPDRS motor score 32.0 compared to 10.4) may explain the differences in distribution of [^11^C]-(*R*)-PK11195 binding and other findings between these two studies.

A recent study in six patients with PD in very early stages (mean UPDRS motor score 7.2) confirmed the results of increased striatal [^11^C]-(*R*)-PK11195 binding as by Gerhard et al. This study also included patients with dementia with Lewy bodies, in whom widespread cortical microglial activation was seen in addition to subcortical changes [33].

Edison and colleagues investigated the relationship between microglial activation, amyloid deposition and glucose metabolism in patients with Parkinson’s disease with and without dementia [34]. The PD patients with dementia showed widespread microglia activation in basal ganglia and cortical regions, which was only slightly more pronounced than in the PD patients without dementia. Interestingly, in the demented Parkinson’s disease patients an inverse correlation between cortical microglia activation and scores on the Mini Mental State examination, a neuropsychological test, could be demonstrated as well as a significant overlap with regions in which glucose metabolism was decreased.

In contrast to studies referenced above Koshimori and colleagues recently published data that did not clearly show an increase of TSPO expression in Parkinson’s disease patients as compared to healthy controls, using the second generation TSPO tracer [^18^F]-FEPPA [35]. However, this study only included the caudate nucleus and putamen as regions of interest and cortical or brainstem signal might have been missed.

The first in vivo study in atypical parkinsonian disorders to demonstrate elevated [^11^C]-(*R*)-PK11195 binding was conducted in five patients with multiple system atrophy (MSA) compared to controls [36]. Here, increased BP in caudate nucleus, putamen, substantia nigra and pons as well as cortical regions was measured.

**Fig. 3:**
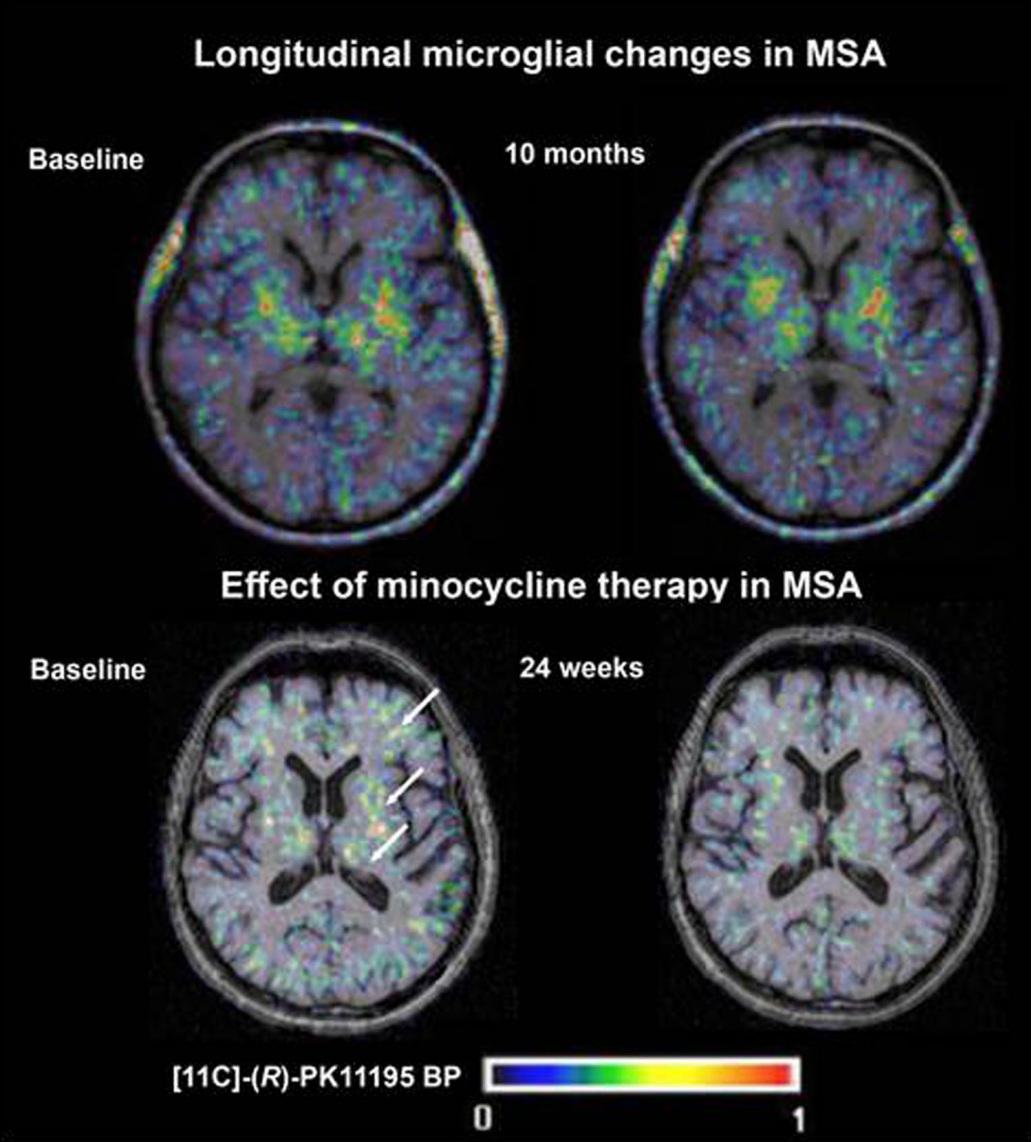
Longitudinal changes in [^11^C]-(*R*)-PK11195 binding potential (BP) seen in a study of minocycline therapy in patients with multiple system atrophy (MSA). Images are transverse BP maps coregistered to structural T1-weighted MR images at baseline (**a**) and after 24 weeks of treatment (**b**). Modified with permission from [37]

In a PET imaging sub-study of a randomized placebo-controlled trial of minocycline in patients with MSA, treatment with minocycline was associated with reductions in [^11^C]-(*R*)-PK11195 binding, but no clinical benefit was shown in the overall study [37] (Fig. 3). However, this study provided proof-of-concept for the use of PK11195 PET in monitoring treatment effects of potential anti-inflammatory agents in neurodegenerative disorders.

Recently one of the new TSPO PET ligands has been used to measure microglial activation in Parkinson’s disease patients treated with AZD3241, a selective and irreversible inhibitor of myeloperoxidase with the putative mode of action of a reduction of sustained neuroinflammation through reduced oxidative stress [38]. Using [^11^C]-PBR28 PET the authors reported a reduction of distribution volume of the radioligand after 4 and 8 weeks of treatment compared to the placebo group where no change was found.

So far, however, there is no published study comparing [^11^C]-PBR28 binding in healthy controls and patients with Parkinson’s disease and it is therefore currently unknown whether ^11^C-PBR28 increases in PD patients follow a similar pattern as in studies with [^11^C]-(*R*)-PK11195.

PET studies using[^11^C]-(*R*)-PK11195 in parkinsonian disorders with tau-pathology were also able to show prominent microglial activation in vivo. In progressive supranuclear palsy, elevated [^11^C]-(*R*)-PK11195 BP was observed in the caudate, putamen, pallidum, substantia nigra, midbrain, thalamus, cerebellum and frontal lobe [39]. In the two patients who were rescanned after 6–10 months the PET signal remained stable.

Similarly, four patients with CBD showed increased [^11^C]-(*R*)-PK11195 BP in caudate, putamen, substantia nigra and frontoparietal cortex [40].

In a recent study [41] the relationship of increased putaminal [^11^C]-(*R*)-PK11195 BP in patients with MSA-P and PSP with MR markers of tissue damage such as apparent diffusion coefficient was investigated; here, microglial activation did not correlate with putaminal water diffusivity.

### Other targets than TSPO to image neuroinflammation with PET

Over the last few years targets involved in the neuroinflammatory process in neurodegenerative disease other than the TSPO have been identified that are currently evaluated using PET ligands.

One very interesting target is the cannabinoid receptor type 2 (CB_2_R), which is involved in peripheral immune system function, and is also upregulated in CNS disorders showing microglial activation [42], including Alzheimer’s disease [43] and animal models of Huntington’s disease [44]. This has led to the development of specific ligands, such as [^11^C]-NE40, which shows binding to the human CB_2_R overexpressed in rodent striatum in preclinical PET studies [45]. First in man to symmetry studies showed a swift brain uptake [46], however, at the time of writing, no clinical data on CB_2_R PET studies in PD have been published.

In addition to microglial activation, proliferation of astrocytes as part is seen in several neurodegenerative conditions including AD, and is associated with increased expression of monoamine oxidase B (MAO B) [47]. Autoradiographic studies in *post-mortem* AD tissue using the radiolabelled selective MAO B inhibitor [^11^C]-L-deprenyl (selegiline) demonstrated specific binding to cortical areas with large numbers of reactive astrocytes and microglia [48]. The deuterium-substituted analog [^11^C]-deuteriodeprenyl (DED) has been utilized as a PET tracer due to its kinetic advantages, including increased sensitivity [49].

So far no studies with this marker have been performed in parkinsonian disorders, but in a study investigating MCI (minimal cognitive impairment) and AD patients, [^11^C]-DED binding was highest in MCI patients compared to healthy controls and AD patients overall. PiB-(a PET marker for amyloid) positive MCI patients had the highest levels of DED binding while AD patients had the least [50]. This raises the possibility that astrocytosis may be an early event in the pathogenesis of AD, a hypothesis supported by a detailed pathological case study in which no relationship was found between post-mortem binding of [^3^H]-PiB, [^3^H]-PK11195 and [^3^H]-L-deprenyl in AD brain tissue [51]. Similarly, in a recent case report of a patient with memory impairment and clinical motor neuron disease increased binding of [^11^C]-DED on PET was suggestive of astrocytosis [52].

Hence, imaging of astrocyte proliferation represents a promising target for quantifying neuroinflammation in several neurodegenerative conditions.

The purinergic receptor P2X7 plays in recognizing α-synuclein, a key protein in Parkinson’s disease [53], and currently PET ligands are been developed for the microglial P2X7 (personal communication Bert Windhorst, Amsterdam).

## Conclusion/summary

[^11^C]-(R)-PK11195 PET is an imaging modality with the ability to demonstrate one aspect of the neuropathology in neurodegenerative disorders in vivo and longitudinally in a minimally invasive fashion.

The studies mentioned above have demonstrated pattern of microglia activation in different parkinsonian syndromes that are in good agreement with the distribution of known neuropathological changes in these disorders. Interestingly the small number of longitudinal studies (between 4 weeks and 24 months) did not show a significant change in the extent of microglia activation although patients progressed clinically.

The successful application of PET imaging for microglia activation enables us to use an “in vivo pathology” approach that also allows to monitor treatment responses in interventional trials.

Despite these interesting possibilities a number of open questions remain. PET only captures one aspect of the neuroinflammatory response, namely the expression of TSPO. It is, however, highly likely that activated microglia performs different functions at different stages of neurodegenerative disorders.

The optimal method of analysis for PET data is also the subject of debate, as the selection of an anatomically defined reference region due to constitutive expression of TSPO within the brain and potentially widespread pathology in neurodegenerative disorders, is difficult. Methods using kinetic analysis to identify a reference tissue cluster have, therefore, been employed in the analysis of [^11^C]-(*R*)-PK11195 PET data in neurodegenerative disease [54]. Lastly, as a [^11^C]-labeled tracer [^11^C]-(*R*)-PK11195 has only a short half-life and necessitates a cyclotron on site for tracer production, which limits wider clinical application.

Currently significant effort goes into the development of novel TSPO ligands for PET imaging in neurodegenerative diseases. Additionally, the recent finding of populations of high- and low-affinity binders for TSPO ligands other than [^11^C]-(*R*)-PK11195 has further complicated the analysis of these data. Thus, studies utilizing such ligands require analysis of TSPO binding affinity to correct for this factor.

Only further clinical evaluations and direct comparisons with [^11^C]-(*R*)-PK11195 will show whether any of the new putative TSPO tracers are indeed superior in the clinical context. As there are known species differences in TSPO binding, in vitro and preclinical animal data do not always directly translate into clinical applications in humans.
